# *Bartonella henselae* Neuroretinitis: A Rare Coinfection in POEMS Syndrome

**DOI:** 10.4274/tjo.galenos.2020.83873

**Published:** 2020-12-29

**Authors:** Mas Edi Putriku Intan Ab Kahar, Julieana Muhammed, Wan Hazabbah Wan Hitam, Azlan Husin

**Affiliations:** 1 Universiti Sains Malaysia School of Medical Sciences, Department of Ophthalmology, Kelantan, Malaysia; 2 Universiti Sains Malaysia School of Medical Sciences, Department of Internal Medicine, Kelantan, Malaysia; 3 Universiti Sains Malaysia Hospital USM, Kelantan, Malaysia

**Keywords:** Bartonella henselae neuroretinitis, POEMS syndrome, bilateral disc swelling

## Abstract

*Bartonella henselae* is a recognized cause of neuroretinitis in cat scratch disease. Meanwhile, polyneuropathy, organomegaly, endocrinopathy, monoclonal gammopathy, skin changes (POEMS) syndrome with Castleman disease (evidence of lymph node hyperplasia), is a chronic debilitating condition that predisposes to various superimposed infections. *B. henselae* neuroretinitis implicated in POEMS syndrome has not been reported previously. A 34-year-old asymptomatic man was referred for an eye assessment. Examination showed visual acuity of 6/18 in the right eye and 6/24 in the left eye. On fundus examination, both eyes exhibited typical features of neuroretinitis (optic disc swelling and incomplete macular star). There was otherwise no vitritis or chorioretinitis. Serology for *B. henselae* revealed high immunoglobulin M (IgM) titer (1:96) indicative of acute disease, and positive immunoglobulin G (IgG) (1:156). He was treated with oral azithromycin for 6 weeks and a short course of oral prednisolone. Subsequently, the visual acuity in both eyes improved with resolution of macular star. However, both optic discs remained swollen.

## Introduction

*Bartonella henselae* is the most common causative agent in neuroretinitis and is transmissible to humans through scratches, bites, or licks from cats or kittens. However, the main vector of transmission between cats and humans is the cat flea (*Ctenocephalia felis*). Cat fleas are found on up to 33% of healthy household pets and strays. However, prior contact with these vectors is not a prerequisite for diagnosis, as Tan et al. reported that only 25% of cases had specific history.^1^

Polyneuropathy, organomegaly, endocrinopathy, monoclonal gammopathy, skin changes (POEMS) syndrome is a rare cause of bilateral optic disc swelling. It was first described in 1938 by Scheinker and was previously known as Takatsuki or Crow-Fukuse syndrome. The prevalence of POEMS was estimated to be 0.3 cases per 100,000 population per year, initially reported in Japanese.^2^ Although polyneuropathy, organomegaly, endocrinopathy, monoclonal gammopathy, and skin changes constitute the acronym POEMS, other salient features were not included in the acronym. Despite an abundance of reports regarding POEMS syndrome in Asian countries, especially Japan, China, and India, it remains relatively rare in the South-east Asia region. In Malaysia, there have been only a few reported cases of POEMS syndrome.^3,4^

This is the first reported case of bilateral *B. hensalea* neuroretinitis in a patient with POEMS syndrome.

## Case Report

A 34-year-old man diagnosed with chronic inflammatory demyelinating polyradiculoneuropathy (CIDP) 2 years earlier was referred for an eye assessment. However, he did not have any visual complaint. He presented initially with progressive bilateral upper and lower limb weakness. After several courses of intravenous immunoglobulin (IVIg), his condition showed minimal improvement.

**Figure 1:**
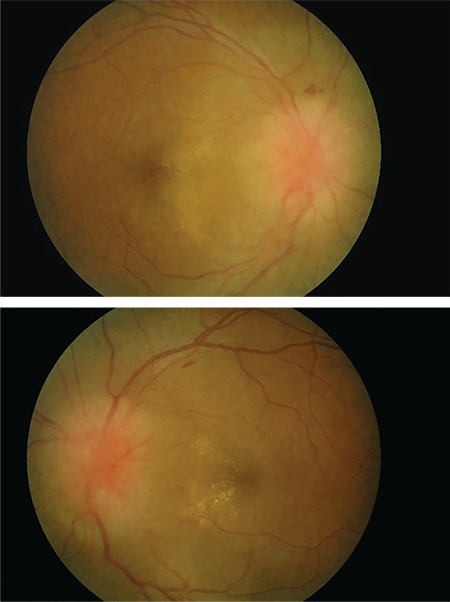
Fundus photography during initial assessment shows bilateral optic disc swelling with disc hemorrhages and incomplete macular star

**Figure 2:**
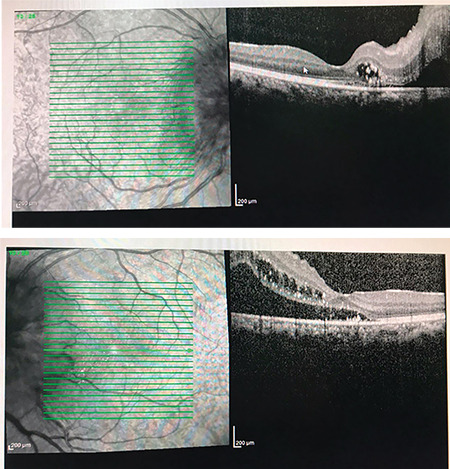
Macular optical coherence tomography shows presence of subretinal fluid, intraretinal edema, and exudates involving the fovea

**Figure 3:**
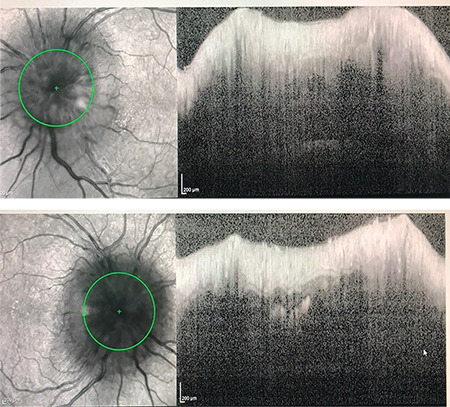
Optical coherence tomography of the bilateral optic nerve head shows diffuse optic nerve edema

**Figure 4:**
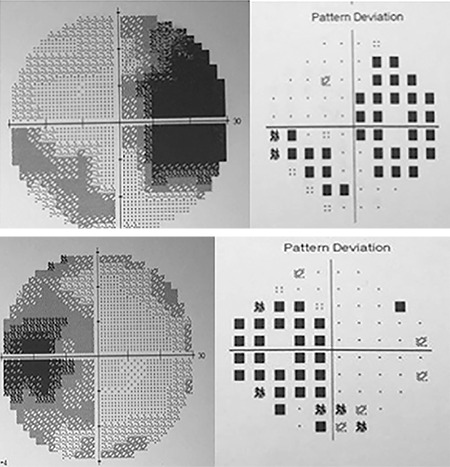
Humphrey visual field grayscale shows bilateral enlarged blind spot

On ocular examination, his best-corrected visual acuity (BCVA) was 6/18 in the right eye and 6/24 in the left eye with absence of relative afferent pupillary defect. Contrast/brightness sensitivity, color vision, and extraocular muscle movements were normal. Anterior segment examination was unremarkable in both eyes. Fundoscopy showed bilateral gross optic disc swelling with splinter hemorrhages. Both maculae were edematous with incomplete stellate macular star (Figure 1). Otherwise, vitritis, dilated tortuous vein, retinal hemorrhages, or chorioretinitis were not seen. Optical coherence tomography of the maculae showed bilateral subretinal fluid collection with exudates extending from the optic disc (Figure 2, 3). Visual field examination revealed a diffusely enlarged blind spot with no central scotoma (Figure 4).

On physical examination, the patient was of medium build with body mass index of 22. He was normotensive with regular heart rate. There was bilateral sensorimotor weakness in the upper limbs and lower limbs until below knee level. Upper limb strength was 3/5 and lower limb strength was 2/5 with reduced plantar reflexes. Other cranial nerve examinations and anal tone were normal.

Infective and immunology screening was performed. *B. henselae* serology showed abnormally high titer of IgM (1:96) and IgG (1:156), which supported the diagnosis of *B. henslelae neuroretinitis*. Other infective screening investigations such as syphilis, viral hepatitis, Mantoux test, and tuberculosis quantiferon tests were negative. Contrast-enhanced computer tomography (CT) scans of the optic nerve and brain to delineate the optic nerve course and evaluate gross structure of the brain were normal. However, the patient refused lumbar puncture.

**Figure 5:**
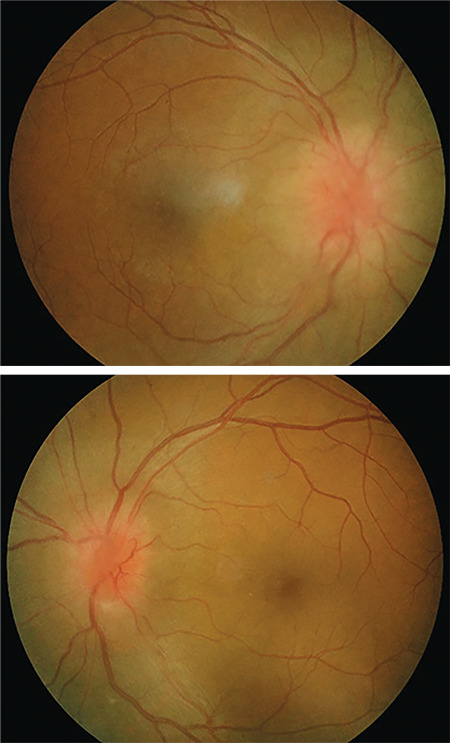
Fundus photography after completed antibiotic and corticosteroid treatment shows resolution of macular star and disc hemorrhages with persistent bilateral optic disc swelling

Oral azithromycin 500 mg daily was given for 6 weeks. Oral prednisolone 60 mg daily (1 mg/kg) was added due to the macular edema. The BCVA in both eyes improved to 6/9 upon treatment completion and the macular edema resolved. However, both optic discs remained swollen (Figure 5).

**Figure 6:**
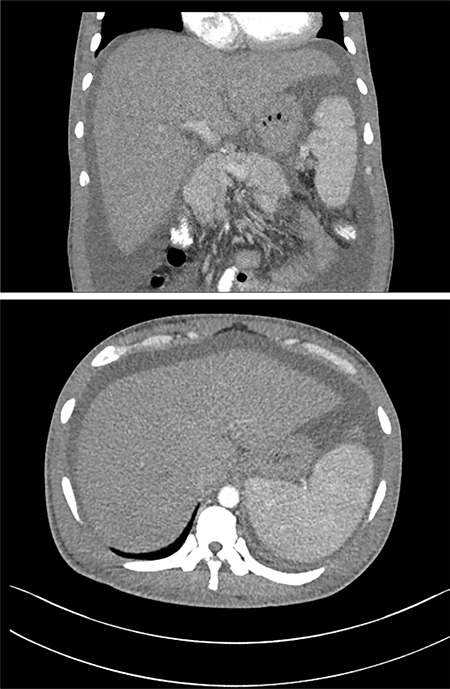
Computed tomography scans of the abdomen showing hepatosplenomegaly

**Figure 7:**
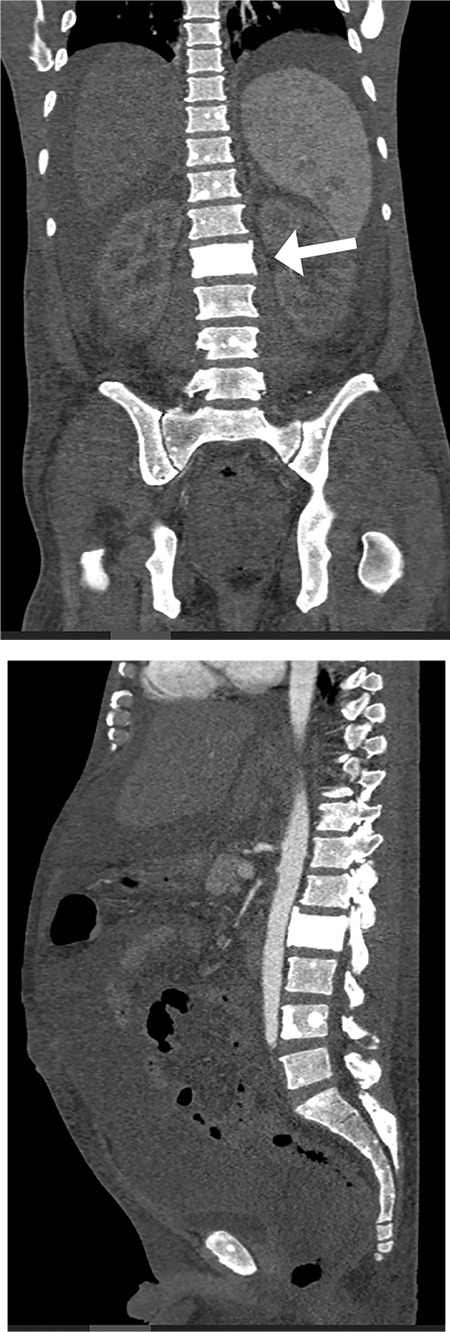
Computed tomography scan of the abdomen and pelvis showing multiple sclerotic bony lesions over the spine with ivory vertebral appearance of L1 (white arrow)

The ocular findings of polyneuropathy prompted further investigations. Follow-up within the next 6 months revealed myriad symptoms that finally led to the diagnosis of POEMS syndrome. Further blood investigations showed microcytic hypochromic anemia with evidence of subclinical hypothyroidism. Systemic work-up revealed hepatomegaly and splenomegaly with recurrent ascites by CT of the hepatobiliary system. Abdominal X-ray showed multiple lytic lesions in the spine and iliac bone (Figure 6,7). Repeated nerve conduction study  revealed segmented demyelination of sensory and motor polyneuropathy that was initially confused for CIDP instead of POEMS syndrome. Electrophoresis of serum protein showed presence of monoclonal IgG-lambda paraprotein. Biopsy of supraclavicular and inguinal lymph nodes swellings pointed to multicentric Castleman disease (MCD). Plasma vascular endothelial growth factor (VEGF) levels, however, was not sent as was not available in the country.

Chemotherapy was subsequently started using vincristine, ifosfamide, carboplastin, dexamethasone; cyclophosphamide, doxorubicin, etoposide, vincristine, prednisone; and cyclophosphamide, doxorubicin, vincristine, prednisone  regimens.

His vision further improved to 6/7.5 bilaterally. However, both optic discs remained persistently swollen. Repeated brain CT with contrast showed no sign of any intracranial lesions, infections, or neurodegenerative changes. Due to financial constraints, the patient refused further investigations including fundus fluorescence angiography or magnetic resonance imaging of the central nervous system.

He subsequently had multiple episodes of extravascular fluid overload and infections due to pneumonia that required multiple hospitalizations. His polyneuropathy worsened and rendered him bedridden. His general condition deteriorated relentlessly, and he finally succumbed to his condition 2 years after diagnosis.

## Discussion

POEMS syndrome is a collection of clinical manifestations resulting from nonmetastatic systemic plasma cell neoplasm. It is now recognized that not all symptoms need to be present to reach the diagnosis. A recent update by Dispenzieri et al.^5^ described the requirement of 2 major mandatory criteria and highlighted the emergence of other important features including Castleman disease, sclerotic bone lesions, elevated VEGF levels, optic disc edema, extravascular volume overload, thrombosis, and abnormal pulmonary function tests (Table 1). Castleman disease is a lymphoproliferative disorder, and can be present concomitantly in 11-50% of patients diagnosed with POEMS syndrome.^6^ This patient fulfilled the minimum of 2 mandatory major criteria with at least 1 major and 1 minor criteria for diagnosis of POEMS syndrome.

Commonly, optic disc swelling with macular exudates arranged in partial or complete star configuration is a typical feature of neuroretinitis. In ocular infection with *B. henselae*, the diagnosis relies on the typical clinical signs, supported with positive serologic testing. We hence propose that due to his immunocompromised state, the patient had concurrent *B. henselae* neuroretinitis with POEMS syndrome. Although cat scratch disease (CSD) is generally a self-limiting disease, treatment of *B. henselae* neuroretinitis may hasten the resolution of macular edema and optic disc swelling, thus favoring good visual outcome. Efficacy in the eradication of *B. henselae* has been observed with doxycycline, rifampicin, gentamicin, cotrimoxazole, and ciprofloxacin. Although the benefit of corticosteroid is still controversial in CSD, a case series of 14 Japanese CSD patients with *B. henselae* neuroretinitis treated with combination antibiotic and corticosteroid therapy showed good visual outcome.^7^

Whilst the pathogenesis of CSD and POEMS syndrome may differ, use of corticosteroids may help control some degree of intraocular inflammation and optic neuropathy by inhibiting migration of inflammatory cells and mediators, especially prostaglandins, leukotrienes, and cytokines (importantly VEGF-A) in the retina, which further reduces vascular leakage and edema.^8^

Optic disc swelling in our patient may have also resulted primarily from POEMS syndrome, which was not diagnosed at primary ocular examination.

Bilateral optic disc swelling is the commonest ocular manifestation in POEMS syndrome. It was present in 30-70% of cases in a large retrospective series evaluating the frequency of POEMS syndrome manifestations.^9^ Less frequently reported ocular signs are retinal hemorrhage, subretinal fluid, macular edema, cotton wool spot, choroidal neovascularization, central retinal artery occlusion, and serous retinal detachment.^10,11^ The ocular findings of bilateral optic disc swelling with macula edema seen as a coinfection with *B. henselae* neuroretinitis as highlighted in our case has not been presented in any previous review.

The pathogenesis of optic disc swelling in POEMS syndrome, however, remains unclear. The possibility of increased intracranial pressure, overproduction of inflammatory mediators with microangiopathy, direct disc infiltrations, or elevated abnormal proteins has been proposed and debated over the years.^12,13^ It has been postulated that as VEGF is a pro-inflammatory and potent angiogenic factor, its overproduction in POEMS syndrome leads to abnormal and leaky endothelial cell proliferation that subsequently leads to plasma leakage. This explains the manifestation of bilateral disc edema in POEMS syndrome despite the absence of increased intracranial pressure, direct compression, or optic nerve infiltrations. Other proinflammatory mediators including cytokines, interleukin-6, interleukin-1b, and tumor necrosis factor-alpha (TNF-α) released by abnormal plasma cells may have caused further vascular permeability, thus leading to worsening of disc edema and systemic fluid overload.^14^

The presence of overlapping features of *B. henselae* neuroretinitis and POEMS syndrome may have been misleading for the treating physician. Therefore, it is crucial that ophthalmologist is aware that chronic non-resolving bilateral disc edema with normal brain imaging and presence of polyneuropathy could point to other multisystemic disease.

Furthermore, it is not surprising that more symptoms that point to POEMS syndrome develop over time. The mean time of diagnosis from initial presentation was reported to be 15 months, ranging from 3 to 120 months according to Dispenzieri et al.^12^

Early diagnosis of POEMS syndrome is, however, crucial in reducing morbidity and also improves survival. Previous studies have reported median survival time after diagnosis of only 165 months and 30 months respectively for POEMS and MCD patients.^15^ A recent study by Wang et al.^16^ reported that most patients died due to cardiorespiratory failure, capillary leakage complications, and infection during the disease course. The total number of presenting features during the initial diagnosis of the disease was insignificant in predicting patient survival. However, finger clubbing and extravascular volume overload were reported as poor prognostic factors.^12,17^ Our patient had these features and passed away 24 months after diagnosis due to severe respiratory tract infection.

In terms of management for POEMS syndrome, there is still no standard guideline established. Radiation is preferred if plasmacytoma is isolated, while chemotherapy is the best option for widespread disease. Successful treatment of POEMS syndrome with systemic anti-VEGF (bevacizumab) and blood stem cell transplantation has remained a controversy. Nakaseko et al.^18^ has proposed the positive role of autologous peripheral blood stem cell transplant in terms of survival and quality of life. However, there is still no clinical trial data with which to conclude the best treatment guidelines for these conditions.

## Conclusion

POEMS syndrome is difficult to diagnose due to its rarity and complexity with multiorgan involvement. Although ocular presentation is an essential part of POEMS syndrome, previous reports mainly highlighted the presence of optic disc edema and scarcely regarded other ocular findings, specifically neuroretinitis. Early diagnosis with meticulous systemic examinations and prompt initiation of treatment is crucial in achieving favorable outcome in POEMS syndrome. Thus, this case report aims to increase awareness regarding POEMS syndrome and possible initial ocular associations, especially among ophthalmologists.

## Figures and Tables

**Table 1:**
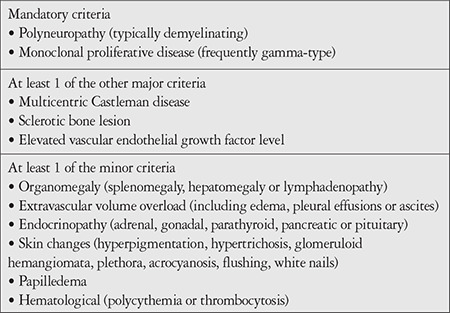
Revised diagnostic criteria for POEMS (polyneuropathy, organomegaly, endocrinopathy, M protein, skin changes) syndrome from Dispenzieri et al.^5^ (2007). Diagnosis of POEMS requires presence of both mandatory criteria, at least 1 of 3 other major criteria, and at least 1 of 6 minor criteria
